# Multicenter Evaluation of the FilmArray Blood Culture Identification 2 Panel for Pathogen Detection in Bloodstream Infections

**DOI:** 10.1128/spectrum.02547-22

**Published:** 2022-12-15

**Authors:** François Caméléna, Gauthier Péan de Ponfilly, Hélène Pailhoriès, Lucas Bonzon, Alexandre Alanio, Thibaut Poncin, Matthieu Lafaurie, François Dépret, Emmanuel Cambau, Sylvain Godreuil, Rachel Chenouard, Alban Le Monnier, Hervé Jacquier, Béatrice Berçot

**Affiliations:** a Département de Bactériologie, Groupe Hospitalier Saint-Louis-Lariboisière-Fernand Widal, Assistance Publique-Hôpitaux de Paris, Paris, France; b Université Paris Cité and Université Sorbonne Paris Nord, INSERM, IAME, Paris, France; c Service de Microbiologie clinique, Groupe hospitalier Paris Saint Joseph, Paris, France; d Institut Micalis UMR 1319, Université Paris-Saclay, INRAe, AgroParisTech, Châtenay Malabry, France; e Laboratoire de Bactériologie, Institut de Biologie en Santé, Centre Hospitalier Universitaire d’Angers, Laboratoire HIFIH, UPRES EA3859, SFR ICAT 4208, Université d’Angers, Angers, France; f Laboratoire de Bactériologie, Centre Hospitalier Universitaire de Montpellier et MIVEGEC, UMR IRD-CNRS-Université de Montpellier, Montpellier, France; g Laboratoire de Parasitologie-Mycologie, Groupe Hospitalier Saint-Louis-Lariboisière-Fernand Widal, Assistance Publique-Hôpitaux de Paris, Paris, France; h Institut Pasteur, Université Paris Cité, CNRS, Unité de Mycologie Moléculaire, UMR2000, Paris, France; i Département des Maladies Infectieuses et Tropicales, Groupe Hospitalier Saint-Louis-Lariboisière-Fernand Widal, Assistance Publique-Hôpitaux de Paris, Paris, France; j Département d’Anesthésie et Réanimation chirurgicale et brûlés, Groupe Hospitalier Saint-Louis-Lariboisière-Fernand Widal, Assistance Publique-Hôpitaux de Paris, Paris, France; k Laboratoire de mycobactériologie spécialisée et de référence, Laboratoire associé du Centre National de Référence des mycobactéries et résistance des mycobactéries aux antituberculeux, Groupe Hospitalier Saint-Louis-Lariboisière-Fernand Widal, Assistance Publique-Hôpitaux de Paris, Paris, France; University Paris-Saclay, AP-HP Hôpital Antoine Béclère, Service de Microbiologie, Institute for Integrative Biology of the Cell (I2BC), CEA, CNRS

**Keywords:** BCID2, BSI, Biofire, bloodstream infection, FilmArray, molecular diagnosis, rapid diagnosis, genotypic identification, resistance gene, sepsis

## Abstract

The FilmArray Blood Culture Identification 2 panel (BCID2; bioMérieux) is a fully automated PCR-based assay for identifying bacteria, fungi, and bacterial resistance markers in positive blood cultures (BC) in about 1 h. In this multicenter study, we evaluated the performance of the BCID2 panel for pathogen detection in positive BC. Conventional culture and BCID2 were performed in parallel at four tertiary-care hospitals. We included 152 positive BC—130 monomicrobial and 22 polymicrobial cultures—in this analysis. The BCID2 assay correctly identified 90% (88/98) of Gram-negative and 89% (70/79) of Gram-positive bacteria. Five bacterial isolates targeted by the BCID2 panel and recovered from five positive BC, including three polymicrobial cultures, were missed by the BCID2 assay. Fifteen isolates were off-panel organisms, accounting for 8% (15/182) of the isolates obtained from BC. The mean positive percent agreement between the BCID2 assay and standard culture was 97% (95% confidence interval, 95 to 99%), with agreement ranging from 67% for Candida albicans to 100% for 17 targets included in the BCID2 panel. BCID2 also identified the *bla*_CTX-M_ gene in seven BC, including one for which no extended-spectrum β-lactamase (ESBL)-producing isolate was obtained in culture. However, it failed to detect ESBL-encoding genes in three BC. Two of the 18 *mecA/C* genes detected by the BCID2 were not confirmed. No carbapenemase, *mecA/C*, or MREJ targets were detected. The median turnaround time was significantly shorter for BCID2 than for culture. The BCID2 panel may facilitate faster pathogen identification in bloodstream infections.

**IMPORTANCE** Rapid molecular diagnosis combining the identification of pathogens and the detection of antibiotic resistance genes from positive blood cultures (BC) can improve the outcome for patients with bloodstream infections. The FilmArray BCID2 panel, an updated version of the original BCID, can detect 11 Gram-positive bacteria, 15 Gram-negative bacteria, 7 fungal pathogens, and 10 antimicrobial resistance genes directly from a positive BC. Here, we evaluated the real-life microbiological performance of the BCID2 assay in comparison to the results of standard methods used in routine practice at four tertiary care hospitals.

## INTRODUCTION

Bloodstream infections (BSI) and sepsis are leading causes of morbidity and mortality, and they constitute a major public health concern, with an estimated burden of 1,200,000 episodes annually in Europe (1). Timely, effective antimicrobial treatment is crucial, as delays in treatment initiation are associated with a poorer outcome, particularly in patients developing septic shock (2, 3). The increase in antimicrobial resistance has led to a decrease in the efficacy of empirical antibiotic treatment (4, 5). Rapid and accurate diagnosis of the causal pathogens, and also the identification of antibiotic resistance genes, can improve the outcome for patients with BSI by allowing more targeted therapy, which is becoming ever more important with the increase in antimicrobial drug resistance (6, 7). Blood culture (BC) in a microbiology laboratory remains the gold standard technique for identifying the pathogens responsible for BSI. This approach includes culture-based methods for pathogen identification and antimicrobial drug susceptibility testing (AST). These methods yield results 24 to 48 h after the obtainment of a positive BC, and this delay may be critical in the management of patients with sepsis (8). It is possible to shorten the time to BSI pathogen identification by using matrix-assisted laser desorption ionization–time of flight mass spectrometry (MALDI-TOF MS)-based approaches, with identification directly on positive BC or after a short period of subculture on solid medium (9, 10). However, these methods are subject to several limitations, including major time-consuming changes in laboratory workflows. Molecular assays based on multiplex PCR or microarrays have been marketed in recent years for the identification of bacterial and fungal pathogens from positive BC within 1 to 4 h, shortening both turnaround and hands-on times (6, 10). The FilmArray Blood Culture Identification 2 panel (BCID2; Biofire; bioMérieux, Marcy l’Etoile, France), an updated version of the original BCID, is a fully automated microbiological diagnostic assay based on nested multiplex PCR analysis that can detect 11 Gram-positive bacteria, 15 Gram-negative bacteria, 7 fungal pathogens, and 10 antimicrobial resistance genes (see below). The aim of this multicenter study was to evaluate the real-life microbiological performance of the BCID2 assay for identifying bacteria and detecting resistance genes, through comparison with the results of standard methods used in routine practice.

## RESULTS

### Concordance and discrepancy between the BCID2 panel and culture for microorganism identification.

In total, 152 positive BC were included in the analysis: 70% (106/152) corresponded to BacT/alert FA plus bottles and 30% (46/152) to BacT/alert FN plus bottles. We obtained 51% (78/152) of the positive BC from the Saint-Louis-Lariboisière-Fernand Widal Hospital Group, 19% (29/152) from Paris Saint-Joseph Hospital Group, 16% (24/152) from Angers University Hospital, and 14% (21/152) from Montpellier University Hospital. The 152 positive BC yielded 130 monomicrobial and 22 polymicrobial cultures. The identification results for pathogens targeted by the BCID2 panel are shown in Table 1, and the details of BC giving discrepant BCID2 and standard culture results are provided in Table 2. Organisms detected in polymicrobial BC are presented in Table S1 of the supplemental material.

**TABLE 1:** Concordance between the BCID2 panel and culture for each microorganism identification target^*a*^

Organism	No. of samples with BCID2/culture result of:				% PPA (95% CI)	% NPA (95% CI)	% OPA (95% CI)	*k* (95% CI)
	+/+	+/−	−/+	−/−				
Acinetobacter calcoaceticus*-baumannii* complex	0	0	0	152		100 (98–100)	100 (98–100)	
Bacteroides fragilis	5	0	0	147	100 (48–100)	100 (98–100)	100 (98–100)	1

*Enterobacterales*	64	0	3	85	96 (87–99)	100 (96–100)	98 (95–100)	0.96 (0.92–1)
Enterobacter cloacae complex	13	0	0	139	100 (75–100)	100 (98–100)	100 (98–100)	1
Escherichia coli	31	0	1	120	97 (84–99)	100 (97–100)	99 (97–100)	0.98 (0.94–1)
Klebsiella aerogenes	3	0	0	149	100 (29–100)	100 (98–100)	100 (98–100)	1
Klebsiella oxytoca	0	0	0	152		100 (98–100)	100 (98–100)	
Klebsiella pneumoniae group	8	0	0	144	100 (63–100)	100 (98–100)	100 (98–100)	1
Proteus spp.	3	0	0	149	100 (29–100)	100 (98–100)	100 (98–100)	1
Salmonella	1	0	0	151	100 (0–100)	100 (98–100)	100 (98–100)	1
Serratia marcescens	5	0	0	147	100 (48–100)	100 (98–100)	100 (98–100)	1

Haemophilus influenzae	0	0	0	152		100 (98–100)	100 (98–100)	
Neisseria meningitidis	0	0	0	152		100 (98–100)	100 (98–100)	
Pseudomonas aeruginosa	13	0	0	139	100 (75–100)	100 (98–100)	100 (98–100)	1
Stenotrophomonas maltophilia	2	0	0	150	100 (16–100)	100 (98–100)	100 (98–100)	1
Enterococcus faecalis	5	0	0	147	100 (48–99)	100 (98–100)	100 (98–100)	1
Enterococcus faecium	13	0	1	138	93 (66–99)	100 (98–100)	99 (97–100)	0.96 (0.88–0.98)
Listeria monocytogenes	1	0	0	151	100 (0–100)	100 (98–100)	100 (98–100)	1

Staphylococcus spp.	32	1	0	119	100 (89–100)	99 (95–100)	99 (97–100)	0.98 (0.94–0.98)
Staphylococcus aureus	5	0	0	147	100 (48–100)	100 (98–100)	100 (98-100)	1
Staphylococcus epidermidis	19	3	0	130	100 (82–100)	98 (94–100)	98 (94–100)	0.92 (0.82–0.98)
Staphylococcus lugdunensis	0	1	0	151		99 (96–100)	99 (97–100)	0

Streptococcus spp.	14	1	1	136	93 (68–100)	99 (96–100)	99 (96–100)	0.93 (0.83–1)
Streptococcus agalactiae	1	0	0	151	100 (0–100)	100 (98–100)	100 (98–100)	1
Streptococcus pneumoniae	0	0	0	152	100 (40–100)	100 (98–100)	100 (98-100)	
Streptococcus pyogenes	4	0	0	148		100 (98–100)	100 (98–100)	1

Candida albicans	2	0	1	149	67 (9–99)	100 (98–100)	99 (97–100)	0.80 (0.41–1)
Candida auris	0	0	0	152		100 (98–100)	100 (98–100)	
Candida glabrata	0	0	0	152		100 (98–100)	100 (98–100)	
Candida krusei	1	0	0	151	100 (0–100)	100 (98–100)	100 (98–100)	1
Candida parapsilosis	0	0	0	152		100 (98–100)	100 (98–100)	
Candida tropicalis	0	0	0	152		100 (98–100)	100 (98–100)	
Cryptococcus neoformans	0	0	0	152		100 (98–100)	100 (98–100)	

a PPA, positive percent agreement; NPA, negative percent agreement; OPA, overall percent agreement; *k*, Cohen’s kappa; CI, confidence interval.

**TABLE 2:** Discrepancy of pathogen identification between the BCID2 panel and culture^*a*^

Sample no.	Bottle type	Gram-staining result	Culture result	BCID2 result
17	Aerobic	GNR	**C. freundii**	None
21	Aerobic	GPCC, GNR	S. epidermidis, **C. freundii**	Staphylococcus spp., S. epidermidis
25	Aerobic	GPCC, GPCP	E. faecalis, S. aureus	E. faecalis, Staphylococcus spp., **S. epidermidis**, S. aureus
34	Anaerobic	GNR	S. marcescens	*Enterobacterales*, S. marcescens, **Staphylococcus** **spp.**
58	Aerobic	GPCC, GPCP	E. faecium, S. epidermidis	E. faecium, Staphylococcus spp., S. epidermidis, **Streptococcus** **spp.**
75	Aerobic	GNR	E. coli, P. mirabilis, M. morganii, ***S. anginosus***	*Enterobacterales*, E. coli, Proteus spp.
77	Aerobic	GPCC, GPCP	**E. faecium**, S. epidermidis	Staphylococcus spp., S. epidermidis, ***S. lugdunensis***
100	Anaerobic	GNR	**E. coli**	None
108	Aerobic	GPCC, GNR	P. aeruginosa, *S. haemolyticus*	P. aeruginosa, Staphylococcus spp., **S. epidermidis**
144	Anaerobic	GPCC	S. hominis	Staphylococcus spp., **S. epidermidis**
166	Aerobic	Yeast	**C. albicans**	None

a GNR, Gram-negative rod; GPCC, Gram-positive cocci in clusters; GPCP, Gram-positive cocci in pairs or chains. Microorganisms found by only one method are indicated in bold.

A total of 177 bacteria from 152 positive BC were identified in culture, of which 90% (88/98) of the Gram-negative and 89% (70/79) of the Gram-positive bacteria were correctly detected by BCID2. In addition, the BCID2 detected three Staphylococcus epidermidis, one Staphylococcus lugdunensis, one Staphylococcus spp., and one Streptococcus spp. that were not confirmed by standard culture of the positive BC. In addition, five bacterial isolates recovered from five positive BC, including three that were polymicrobial, were missed by the BCID2 assay. These isolates corresponded to one Escherichia coli and two Citrobacter freundii isolates (genera not included in the BCID2 panel but normally detected as *Enterobacterales*), one Streptococcus anginosus isolate (normally detected as Streptococcus spp.), and one Enterococcus faecium isolate. For fungi, three yeasts that grew in culture and were targeted by the BCID2 panel were correctly identified, and one Candida albicans isolate was missed by the BCID2 assay.

Fifteen bacterial or fungal pathogens in 15 positive BC (10%, 15/152), corresponding to 8% (15/182) of the isolates grown from BC, were not targeted by the BCID2 panel. These organisms are listed in Table 3. They were isolated from monomicrobial cultures from BC with a time to positivity of >35 h. Seven of the 15 isolates not targeted by the BCID2 panel were species likely to be BC contaminants. Most of the remaining pathogens were Gram-negative and/or anaerobic bacteria. One isolate of Fusarium spp. not targeted by BCID2 was also found in one BC.

**TABLE 3:** Cultured organisms not targeted by the BCID2 panel

Sample no.	Bottle type	Time to positivity of BC	Culture	Isolate identification
7	Aerobic	36 h	Monomicrobial	*Achromobacter* spp.
13	Aerobic	43 h	Monomicrobial	Capnocytophaga sputigena
14	Aerobic	75 h	Monomicrobial	*Paenibacillus* spp.
32	Aerobic	46 h	Monomicrobial	Sphingomonas paucimobilis
70	Aerobic	54 h	Monomicrobial	Corynebacterium mucifaciens
73	Anaerobic	38 h	Monomicrobial	Gram-positive rod nonidentified by MALDI-TOF MS
76	Anaerobic	119 h	Monomicrobial	Cutibacterium acnes
82	Anaerobic	53 h	Monomicrobial	Capnocytophaga sputigena
89	Anaerobic	48 h	Monomicrobial	Clostridium perfringens
101	Anaerobic	48 h	Monomicrobial	Bacteroides uniformis
124	Aerobic	74 h	Monomicrobial	Moraxella osloensis
140	Aerobic	52 h	Monomicrobial	Campylobacter jejuni
157	Aerobic	52 h	Monomicrobial	Facklamia languida
159	Anaerobic	41 h	Monomicrobial	Eggerthella lenta
165	Aerobic	90 h	Monomicrobial	Fusarium spp.

### Detection of resistance markers targeted by the BCID2 panel.

The results for the resistance genes detected by the BCID2 panel are presented in Table 4. Three CTX-M type extended-spectrum β-lactamase (ESBL)-producing E. coli, two Enterobacter cloacae isolates, and one Klebsiella pneumoniae isolate (all confirmed by AST) were also accurately detected by BCID2. The discrepant results corresponded to one ESBL-producing E. coli for which no target was detected with BCID2 and one sample for which Serratia marcescens and *bla*_CTX-M_ were detected with BCID2 but for which wild-type S. marcescens and AmpC-hyperproducing Citrobacter freundii grew in culture. Additionally, two ESBL-producing E. cloacae isolates, for which BCID2 detected only the E. cloacae without the *bla*_CTX-M_ gene target, were observed, resulting in the failure of the system to predict this phenotype. AST did not reveal an acquired carbapenem resistance phenotype, and no carbapenemase gene was detected with BCID2. A *mecA/C* signal was detected for 18 of the 152 samples tested with BCID2. All these signals were associated with S. epidermidis targets (including one with S. epidermidis and *S. lugdunensis* targets). All these 18 samples yielded positive cultures for Staphylococcus and 15 were concordant with the growth in culture of methicillin-resistant (MR) S. epidermidis. The remaining three samples yielded non-epidermidis staphylococci on culture, which were methicillin susceptible (MS) for two of these samples. The MS phenotype was confirmed by an immunochromatography test performed from colonies (Alere PBP2a SA culture colony test; Abbott, Scarborough, Maine, USA) (11).

**TABLE 4:** Detection results for resistance markers targeted by the BCID2 panel

Phenotype and organism	Resistance marker detected by BCID2^*a*^				No detection of relevant organism by BCID2^*b*^
	*bla*_CTX-M_	*mecA/C*	*mecA/C* and MREJ	No detection	
Presence of ESBL phenotype^*c*^	6			2	1
E. coli	3			0	1
E. cloacae	2			2	0
K. pneumoniae	1			0	0
Absence of ESBL phenotype^*c*^	1			55	2
Methicillin-resistant phenotype^*d*^		16	0	0	6
S. epidermidis		15		0	0
Other *Staphylococcus*^*e*^		1^*f*^		0	6
Methicillin-susceptible phenotype^*d*^		2	0	8	0
S. aureus			0	5	0
S. epidermidis		0		3	0
Other *Staphylococcus*^*e*^		2^*g*^		0	0

a Only if a bacterium allowing the report of the resistance gene was detected. No isolate harbored a phenotype compatible with the presence of carbapenemase genes *vanA*, *vanB*, or *mcr1.1*.

b No detection of a bacterium allowing the report of the resistance gene of interest.

c Based on antimicrobial drug susceptibility testing on *Enterobacterales* isolates.

d Based on antimicrobial drug susceptibility testing on staphylococcal isolates.

e Species other than S. aureus, S. epidermidis, and *S. lugdunensis*.

f *mecA/C* and S. epidermidis detected by BCID2 with a positive culture of methicillin-resistant Staphylococcus haemolyticus.

g *mecA/C* and S. epidermidis detected by BCID2 with a positive culture of methicillin-susceptible Staphylococcus hominis.

### Turnaround times of BCID2 testing and culture results.

The median time to positivity of the BC was 13.9 h (interquartile range [IQR], 10.3 to 19.5 h). The median turnaround time (TAT; i.e., from bottle processing after flagging as positive to the first report of the results) for BCID2 testing was 3.5 h (IQR, 2.4 to 4.8 h). The median TAT for BCID2 testing was significantly shorter than the 25.7 h (IQR, 18.4 to 27.2 h) required for standard culture (*P* < 0.001) and the 27.4 h (IQR, 25.7 to 47.6 h) required to obtain AST results (*P < *0.001). The instrument used for bacterial identification had no impact on the TAT, while AST results obtained with the Vitek2 instrument had a TAT longer than that obtained by disk diffusion (Table S2).

### Detection of pathogen and resistance markers in spiked BC bottles.

Isolates in spiked BC bottles included K. pneumoniae (*n* = 4), E. faecium (*n* = 2), Pseudomonas aeruginosa (*n* = 1), S. aureus (*n* = 1), and Acinetobacter baumannii (*n* = 1) harboring resistance genes, which included *bla*_CTX-M_ (*n* = 2), *bla*_NDM_ (*n* = 3), *bla*_KPC_ (*n* = 1), *bla*_VIM_ (*n* = 2), bla_IMP_ (*n* = 1), *bla*_OXA-48-like_ (*n* = 2), *vanA* (*n* = 1), *vanB* (*n* = 1), and *mecA* and MREJ (*n* = 1). The BCID2 panel identified correctly the nine isolates spiked in four BC bottles and the 14 resistance markers. The characteristics of the isolates and the spiked BC bottles are presented in Table S3.

## DISCUSSION

One of the major challenges in BSI diagnosis is decreasing the time between sampling and bacterial identification (6, 7, 12). Indeed, it generally takes at least 1 or 2 days to obtain identification and AST results following the obtainment of a positive BC. By shortening the time taken to obtain results, it should be possible to decrease the risk of patients being treated inappropriately or unnecessarily with broad-spectrum antimicrobial agents.

In this prospective multicenter study, parallel evaluations of standard culture and BCID2 were performed on 152 positive BC. We found (i) excellent concordance between BCID2 and a traditional workflow and (ii) a significant decrease in turnaround time.

Globally, BCID2 had an excellent overall percent agreement (OPA; 100%) relative to conventional culture for the pathogens present in the panel (Table 1). For all pathogens included in the panel, BCID2 had an overall positive percent agreement (PPA) of 97%, with six microorganisms (two C. freundii [*Enterobacterales* target]), one E. coli ([*Enterobacterales* and E. coli targets], one *S. anginosus* [Streptococcus spp. target], one E. faecium [E. faecium target], and one C. albicans [C. albicans target]) detected by culture alone (Tables 1 and 2). These pathogens have also been reported to remain undetected by BCID2 in other studies, mostly in polymicrobial cultures (13, 14). In our study, only two of the undetected bacteria were isolated from polymicrobial cultures, in which they were found with S. epidermidis detected by BCID2 (Table 2). Off-panel bacteria were detected by culture in 10% (15/152) of the samples. This suggested that the panel includes the vast majority of bacteria causing BSI. This proportion of off-panel microorganisms was lower than reported in previous studies evaluating the first version of the BCID panel (15, 16). Interestingly, these off-panel pathogens included three obligate anaerobic bacteria: Eggerthella lenta, Bacteroides uniformis, which is not detected by the Bacteroides fragilis target, and Clostridium perfringens. As *Bacteroides* and *Clostridium* account for most of the obligate anaerobes identified in BSI, adding pan-*Bacteroides* and pan-*Clostridium* targets to the BCID2 panel would facilitate the rapid identification of these genera, which can cause severe infections if not picked up sufficiently early (17–19). Furthermore, the shape of pathogen(s) on the Gram stain allows the microbiologist to suspect certain pathogens, in particular, fungi and Campylobacter, which are not detected by BCID2.The overall performance of BCID2 was consistent with that reported in other studies (13, 14, 20–22). Holma et al. performed a study on 80 positive clinical BC samples in which the PPA was 99% and the NPA was 100% for all samples (21). The BCID2 assay can also detect pathogens in polymicrobial BC. In our study, 22 positive BC yielded polymicrobial cultures (22/152, 14%) containing two to four different microorganisms. We obtained completely concordant results between BCID2 and culture in 73% (16/22) of the BC containing multiple organisms (Table S1). However, other BCID studies have shown that the presence of multiple organisms favors discordant results between BCID and culture (15, 23, 24).

In addition, there are only a few alternative fully automated assays to the BCID2 panel that can provide broad-spectrum species identification and detect antimicrobial resistance markers in positive BC. Verigene System bloodstream infection tests (Nanosphere, Inc., Northbrook, IL, USA) and the ePlex (GenMark Diagnostics, Carlsbad, CA, USA) provide two or three separate panels for Gram-positive, Gram-negative, and yeast-related organisms and resistance gene targets. The ePlex has a sensitivity of 93 to 100% and a specificity of 97 to 100%, and the Verigene System has a sensitivity of 50 to 100% and a specificity of 99 to 100%, depending on the organism targeted (25, 26). Good performance has also been reported for the Unyvero Blood Culture molecular assay (Curetis, Holzgerlingen, Germany), with a global sensitivity of 97% and a specificity of 99 to 100% with results in around 5 h (27). Recently, a test using a magnetic resonance-based technology (T2Candida and T2Bacteria panels; T2 Biosystem, Lexington, MA, USA) was developed that detects microbial cells directly within whole blood, skipping the BC incubation step. This new test showed a higher sensitivity than BC with a shorter TAT (only a few hours between sampling and results), but the T2Bacteria panel is limited to detection of six bacterial species (28, 29).

The BCID2 panel can detect key genetic markers underlying cephalosporin and carbapenem resistance in Gram-negative bacteria, which is of great potential importance for antimicrobial drug stewardship. The detection of the *bla*_CTX-M_ gene, the most prevalent ESBL-encoding gene worldwide, and major carbapenemase genes (*bla*_KPC_, *bla*_NDM_, *bla*_OXA-48_, and *bla*_VIM_) by BCID2 may decrease the time to optimal and effective treatment, particularly in institutions with high rates of resistance in Gram-negative bacteria. In our study, the BCID2 panel detected seven *bla*_CTX-M_ genes, six of which were confirmed by antimicrobial drug susceptibility testing. However, three ESBL-producing isolates were not detected, including one ESBL-producing E. coli isolate for which the E. coli target was not detected and two E. cloacae strains for which the bacterial target was detected by BCID2 without detection of the *bla*_CTX-M_ gene. These results can be explained either by a lack of detection of the *bla*_CTX-M_ gene or by the production of a non-CTX-M enzyme. Indeed, the ESBL-encoding genes of E. cloacae are more diverse than those of E. coli or K. pneumoniae. In France, up to 40% of ESBL-encoding genes in E. cloacae isolates are non-CTX-M-encoding genes (30). This rate is higher than that in E. coli or K. pneumoniae, in which 95% of ESBL are encoded by a *bla*_CTX-M_ gene. These findings suggest that molecular tests may have a limited ability to predict ESBL phenotype when an E. cloacae target is detected. Unfortunately, no molecular analysis was performed to identify the ESBL-encoding genes in either of the E. cloacae isolates. Furthermore, no MR S. aureus was detected with either BCID2 or in culture, but given published BCID data, we would expect BCID2 to detect this resistance well (16, 24). BCID2 correctly identified all resistance markers in spiked BC, indicating that the assay could detect resistance genes in polymicrobial BC.

The median TAT for organism identification by BCID2 was significantly shorter than that for conventional culture methods, with identification results generally available 18.5 h earlier for the BCID2 assay. The difference between the median TAT (3.5 h) of the BCID2 results compared to the duration of the analysis (~1 h) corresponded to the time to perform the BCID2 in parallel to the standard process, which can be impacted by the number of positive blood cultures daily. This result is similar to those of previous studies on the first version of the BCID panel and for other fully automated assays (25, 26, 31). Alternative methods for rapid identification using a bacterial pellet from positive BC or short-term bacterial incubation on a solid medium have been reported, but these require a reorganization of laboratory workflow to be routinely performed (32). Rapid identification can affect antimicrobial drug prescription, particularly in conjunction with real-time antibiotic stewardship recommendations (33), as demonstrated by the decrease in median time to optimal therapy in both pediatric and adult populations (34, 35).

The principal limitation of this study was the small number of samples positive for some of the pathogens included in the BCID2 panel. In particular, A. baumannii, Haemophilus influenzae, Streptococcus pneumoniae, and Neisseria meningitidis were not detected, and the small number of cases of fungemia (*n *= 5) in our study made it impossible to draw firm conclusions about the performance of the BCID2 panel for detecting fungal pathogens. Further studies are needed to evaluate the performance of the BCID2 panel for those pathogens. We were also unable to confirm discrepant results due to the absence of conserved BC materials for supplementary tests. Another limitation of this study was the absence of cultured organisms displaying carbapenem or glycopeptide resistance in positive BC from patients. Other studies, with a higher frequency of resistant bacteria, will be required for clinical evaluations of these specific targets. However, simulated BC showed that BCID2 detected resistant markers, including variants of carbapenemase genes. Finally, the clinical impact of the BCID2 panel on antibiotic treatment was not evaluated here. A few studies have suggested that it may be possible to use BCID2 for antibiotic stewardship purposes, but further studies are required to address this point (14, 21, 22, 36).

## MATERIALS AND METHODS

### Population and specimen collection.

This study was conducted from March to October 2020 at four tertiary care hospitals distributed in three distinct geographic regions of France (Saint-Louis-Lariboisière-Fernand Widal Hospital Group, AP-HP, Paris; Paris Saint-Joseph Hospital Group, Paris; Angers University Hospital, Angers; and Montpellier University Hospital, Montpellier). BC meeting the following criteria were excluded from the study: previous BC obtained within the last 7 days and included in the study, or no AST performed for a bacterial isolate from a positive BC or invalid BCID2 results (*n* = 3, invalidity rate of 1.9% [3/155]). BC were included on weekdays during the open hours of each laboratory, at the discretion of the clinical microbiologists responsible for the study at each center. Conventional culture and BCID2 were performed in parallel, and the BCID2 result was not communicated to the prescribing physician.

### Standard microbiology methods.

FA plus and FN plus BC bottles were incubated in a BacT/Alert VIRTUO system (bioMérieux, France) at 35°C. For bottles considered positive, the contents were fixed for microscopy and Gram staining and were then cultured for isolation of the bacteria and fungi responsible for BSI, in accordance with the current French Guidelines for Medical Microbiology (REMIC v6 2018) (37). One drop (50 μL) from each BC bottle was used to inoculate each of two sheep blood agar plates and one chocolate agar plate (bioMérieux). These plates were incubated at 35°C in ambient air, under an anaerobic atmosphere, and under an atmosphere containing 5% CO_2_, respectively. If Gram-negative rods were detected by Gram staining, a drop of the BC was used to inoculate a Drigalski agar plate. If a polymicrobial BSI was identified by Gram staining, then colistin-nalidixic acid sheep blood and Drigalski agar plates were added. If a fungal element was detected by Gram staining, a Sabouraud agar plate was added. Bacteria were identified with the Vitek2 system (bioMérieux, Marcy l’Etoile, France) and/or MALDI-TOF MS (Microflex LT [Bruker Daltonics, Bremen, Germany] or Vitek MS [bioMérieux, Marcy l’Etoile, France]), depending on the hospital laboratory. AST was performed by the disk-diffusion method, or with the automated Vitek2 system (bioMérieux, France), depending on the laboratory procedure used in the hospital concerned and/or the species identified, and the results were interpreted according to EUCAST recommendations. Detection of ESBL production was based on AST results and additional synergy tests if necessary (Mastdiscs D68C; Mast Group, Bootle, United Kingdom).

### FilmArray BCID2 panel testing.

The BCID2 test is CE marked for *in vitro* diagnosis and is FDA cleared. BCID2 testing was performed according to the manufacturer’s instructions. Briefly, the contents of the BC bottle were homogenized, and 200 μL was withdrawn and added directly to a dilution buffer, which was then injected into the FA-PP cartridge. Nucleic acid extraction, amplification, detection, and analysis were performed automatically within the cartridge, with a total run time of about 1 h. BCID2 tests were run on the FilmArray V2.0 system. Bacteria, fungi, and resistance genes were analyzed qualitatively with classification as “detected” or “not detected.” The panel includes 26 bacterial targets (Enterococcus faecalis, Enterococcus faecium, Listeria monocytogenes, Staphylococcus spp., Staphylococcus aureus, Staphylococcus epidermidis, Staphylococcus lugdunensis, Streptococcus spp., Streptococcus agalactiae, Streptococcus pneumoniae, Streptococcus pyogenes, Acinetobacter calcoaceticus*-baumannii* complex, Bacteroides fragilis, Haemophilus influenzae, Neisseria meningitidis, Pseudomonas aeruginosa, Stenotrophomonas maltophilia, *Enterobacterales*, Enterobacter cloacae complex, Escherichia coli, Klebsiella aerogenes, Klebsiella pneumoniae group, Klebsiella oxytoca, Proteus spp., Salmonella spp., and Serratia marcescens), seven fungal targets (Candida albicans, Candida auris, Candida glabrata, Candida krusei, Candida parapsilosis, Candida tropicalis, Cryptococcus neoformans*/gattii*), and 10 antimicrobial resistance genes (ESBL *bla*_CTX-M_, carbapenemases *bla*_KPC_, *bla*_NDM_, *bla*_IMP_, *bla*_OXA-48-like_, and *bla*_VIM_, vancomycin resistance *vanA/B*, methicillin resistance *mecA/C* and MREJ, and colistin resistance *mcr-1*).

### Spiking of blood culture bottles.

The BC bottles were spiked following the EUCAST RAST QC protocol (www.eucast.org). In brief, a bacterial suspension of 100 to 500 CFU was suspended in 5 mL of sterile sheep blood and inoculated in an aerobic blood culture bottle. The bottles were incubated in the Virtuo instrument until the instrument signaled positive growth, and the specimen underwent subsequently analysis using the BCID2 test as described above. The clinical isolates used to spike BC were characterized by whole-genome sequencing.

### Statistical analysis.

Positive percent agreement (PPA), negative percent agreement (NPA), overall percent agreement (OPA), Cohen’s κ coefficient, and two-tailed 95% confidence intervals were calculated by comparing the results for conventional culture (reference method) with those for BCID2 exclusively for the bacterial and fungal pathogens present in the BCID2 panel. A Wilcoxon signed-rank test was used to compare the turnaround time (TAT) between the two methods.

### Ethics statement.

This study was performed in accordance with the Declaration of Helsinki. The samples used in this study were collected during routine patient management without the need for additional sampling, and the BCID2 result was not communicated to the prescribing physician. As such, this study did not require ethics committee approval or informed consent from the patients.
