# Supplementary material for: Multicenter Evaluation of the FilmArray Blood Culture Identification 2 Panel for Pathogen Detection in Bloodstream Infections
Source: Microbiol Spectr. 2022 Dec 15;11(1):e02547-22. doi: 10.1128/spectrum.02547-22 (PMC9927563; doi:10.1128/spectrum.02547-22)
Supplement: Supplemental file 1 — Supplemental material. Download spectrum.02547-22-s0001.pdf, PDF file, 0.1 MB [file spectrum.02547-22-s0001.pdf]

Appendix

Supplementary Table S1. Results of BCID2 panel and culture for polymicrobial BC.

Sample No.	Bottle type	Time to positivity of BC	Culture results	BCID2 results
11	anaerobic	12 h	*E. coli, E. feacalis*	*Enterobacterales, E. coli, E. feacalis*
21	aerobic	8 h	***C. freundii***, *S. epidermidis*	*Staphylococcus* spp., *S. epidermidis*
22	aerobic	4 h	*S. marcescens, P. aeruginosa*	*Enterobacterales, S. marcescens, P. aeruginosa*
25	aerobic	12 h	*E. faecalis, S. aureus*	*E. faecalis, Staphylococcus* spp., ***S. epidermidis***, *S. aureus*
31	aerobic	14 h	*E. cloacae, S. epidermidis, S. hominis*	*E. cloacae, Staphylococcus* spp., *S. epidermidis*
46	aerobic	12 h	*E. faecalis, S. capitis*	*E. faecalis, Staphylococcus* spp.
49	aerobic	21 h	*E. faecalis, S. epidermidis*	*E. faecalis, Staphylococcus* spp., *S. epidermidis*
50	aerobic	14 h	*E. faecium, S. haemolyticus*	*E. faecium, Staphylococcus* spp.
56	aerobic	10 h	*P. aeruginosa, E. faecium, S. epidermidis*	*P. aeruginosa, E. faecium, Staphylococcus* spp., *S. epidermidis*
57	anaerobic	4 h	*K. pneumoniae, E. cloacae, S. anginosus*	*Enterobacterales, K. pneumoniae, E. cloacae, Streptococcus* spp.
58	aerobic	12 h	*E. faecium, S. epidermidis, S. haemolyticus*	*E. faecium, Staphylococcus* spp., *S. epidermidis*, ***Streptococcus* spp.**
75	aerobic	2 h	*E. coli, P. mirabilis, M. morganii*, ***S. anginosus***	*Enterobacterales, E. coli, Proteus* sp.
77	aerobic	13 h	***E. faecium***, *S. epidermidis*	*Staphylococcus spp., S. epidermidis, S. lugdunensis*
108	aerobic	15 h	*P. aeruginosa, S. haemolyticus*	*P. aeruginosa, Staphylococcus* spp., ***S. epidermidis***
118	aerobic	16 h	*E. faecium, S. aureus*	*E. faecium, Staphylococcus* spp., *S. aureus*
119	aerobic	10 h	*E. cloacae, E. faecium*	*Enterobacterales, E. cloacae, E. faecium*
120	aerobic	7 h	*C. freundii, S. marcescens*	*Enterobacterales, S. marcescens*
160	aerobic	12 h	*E. coli, E. faecium*	*Enterobacterales, E. coli, E. faecium*
161	anaerobic	11 h	*K. pneumoniae, S. mitis*	*Enterobacterales, K. pneumoniae, Streptococcus* spp.
162	anaerobic	46 h	*P. aeruginosa, S. haemolyticus*	*P. aeruginosa, Staphylococcus* spp.
163	aerobic	13 h	*S. epidermidis, S. mitis*	*Staphylococcus* spp., *S. epidermidis, Streptococcus* spp.
164	aerobic	13 h	*E. faecium, S. haemolyticus*	*E. faecium, Staphylococcus* spp.

Microorganisms found by only one method are indicated in bold.

Supplementary Table S2. Turnaround time for positive blood culture.

Turnaround time (TAT)	Median (IQR)
TAT to pathogen identification by VITEK-MS (h)	25.8 (21.9 - 26.8)
TAT to pathogen identification by Microfex LT (h)	25.6 (17.3 - 27.5)
TAT to AST results by VITEK2 (h)	46.4 (31.7 - 48,6)
TAT to AST results by disc diffusion (h)	26.4 (25.5 - 30.4)
TAT to BCID2 results (h)	3.5 (2.4 - 4.8)

AST : Antimicrobial susceptibility testing, BCID2 : Blood culture identification 2 panel

Supplementary Table S3. Results of BCID2 panel for spiked BC bottles.

Bottle no.	Isolate (resistance determinant)[a]	BCID2 results
1	*K. pneumoniae* (*bla*$_{\text{VIM-1}}$), *E. faecium* (*vanB*)	*Enterobacterales*, *K. pneumoniae*, *E. faecium*, *bla*$_{\text{VIM}}$, *vanA/B*
2	*K. pneumoniae* (*bla*$_{\text{CTX-M-15}}$, *bla*$_{\text{OXA-48}}$, *bla*$_{\text{NDM-1}}$), *S. aureus* (*mecA*)	*Enterobacterales*, *K. pneumoniae*, *Staphylococcus* spp., *S. aureus*, *bla*$_{\text{CTX-M}}$, *bla*$_{\text{OXA-48-like}}$, *bla*$_{\text{NDM}}$, *mecA/C* and MREJ
3	*K. pneumoniae* (*bla*$_{\text{CTX-M-15}}$, *bla*$_{\text{OXA-181}}$, *bla*$_{\text{NDM-5}}$), *P. aeruginosa* (*bla*$_{\text{VIM-4}}$)	*Enterobacterales*, *K. pneumoniae*, *P. aeruginosa*, *bla*$_{\text{CTX-M}}$, *bla*$_{\text{OXA-48-like}}$, *bla*$_{\text{NDM}}$, *bla*$_{\text{VIM}}$
4	*K. pneumoniae* (*bla*$_{\text{KPC-2}}$), *A. baumannii (bla*$_{\text{NDM-1}}$, *bla*$_{\text{IMP-37}}$*)*, *E. faecium* (*vanA*)	*Enterobacterales*, *K. pneumoniae*, *A. baumannii*, *E. faecium*, *bla*$_{\text{KPC}}$, *bla*$_{\text{IMP}}$, *bla*$_{\text{NDM}}$, *vanA/B*

[a]β-lactamase-encoding genes were identified by whole-genome sequencing.
